# A nuanced role of the small loop of hepatitis B virus small envelope protein in virion morphogenesis and secretion

**DOI:** 10.1186/s12929-021-00780-0

**Published:** 2021-12-01

**Authors:** Chih-Hsu Chang, Shu-Fan Chou, Chiaho Shih

**Affiliations:** 1 Graduate Institute of Microbiology, College of Medicine, National Taiwan University, Taipei, Taiwan; 2 Institute of Biomedical Sciences, Academia Sinica, Taipei, Taiwan; 3 Department of Microbiology, Harvard Medical School, Boston, MA USA; 4 Graduate Institute of Medicine, Kaohsiung Medical University, Kaohsiung, Taiwan

**Keywords:** Hepatitis B virus, HBV, Virion secretion, Envelope protein, Small loop, Proline substitution

## Abstract

**Background:**

The virion secretion mechanism of human hepatitis B virus (HBV) remains to be investigated. In our current study, we characterized a reverse transcriptase mutant, which changed from the YMDD motif to YMHA. We noted that this mutant YMHA secreted no virions in the medium. Because of the overlapping open reading frame (ORF) between the polymerase and the envelope genes, the lack of virion secretion is likely due to corresponding concurrent mutations in a small loop of the envelope protein (HBsAg, HBV surface antigen). In literature, small loop mutations are thought to affect virion secretion of hepatitis delta virus (HDV), but not HBV.

**Methods:**

Here, we revisited the relationship between the small loop and virion secretion by site-directed mutagenesis and native agarose gel electrophoresis.

**Results:**

A proline substitution at residue 196 or 198 in the small loop blocked both HBV genome-containing and genome-free virion secretion, but not the secretion of 22-nm HBsAg subviral particles. Surprisingly, a leucine substitution at residue 196 enhanced genome-containing virion secretion. It is also intriguing that a proline-197, sandwiched by residue 196 and 198, exhibited no apparent defect in secreted virions, with or without containing an HBV genome. By complementation assay, we demonstrated that the wild type small envelope protein alone is sufficient to rescue the virion secretion defect of a small loop mutant M198P.

**Conclusions:**

The effect of the small loop mutation of HBV small envelope protein on virion secretion is position-dependent. It warrants further investigation how the small loop of HBsAg plays a subtle role in HBV morphogenesis and secretion of virions with or without containing an HBV genome.

**Supplementary Information:**

The online version contains supplementary material available at 10.1186/s12929-021-00780-0.

## Background

Hepatitis B virus (HBV) is a human hepatotropic DNA virus (hepadnavirus) [1, 2]. Chronic active hepatitis B patients have a higher risk to develop liver cirrhosis, liver failure and hepatocellular carcinoma. At present, no treatment can effectively eradicate the virus from chronic patients [3]. Therefore, long term treatment is required to inhibit viral reactivation from the HBV reservoir. Some HBV patients are coinfected with hepatitis delta virus (HDV) leading to more severe liver diseases [4]. Like a parasite virus to HBV, HDV is dependent on the envelope protein of HBV for viral entry and virion secretion.

HBV surface antigen (HBsAg) is a group of structurally related envelope proteins, including large (L), middle (M) and small (S) envelope. In addition to the envelope protein, HBV also encodes core (capsid) protein for nucleocapsid, polymerase (pol) for pregenomic RNA encapsidation, reverse transcription, and DNA synthesis. In the cell culture transfection system, a number of viral and subviral particles can be detected in the medium. The best known subviral particles are the non-infectious HBsAg particles around 22-nm in diameter. Enveloped viral particles in the extracellular compartment include genome-containing virions and genome-free empty virions. Empty virions can be detected in patients and in cell culture [5–11]. Non-enveloped particles in the medium include genome-containing nucleocapsids and genome-free empty capsids. The mechanisms for the assembly and release of these various viral and subviral particles are highly complicated, and remain to be elucidated.

**Fig. 1:**
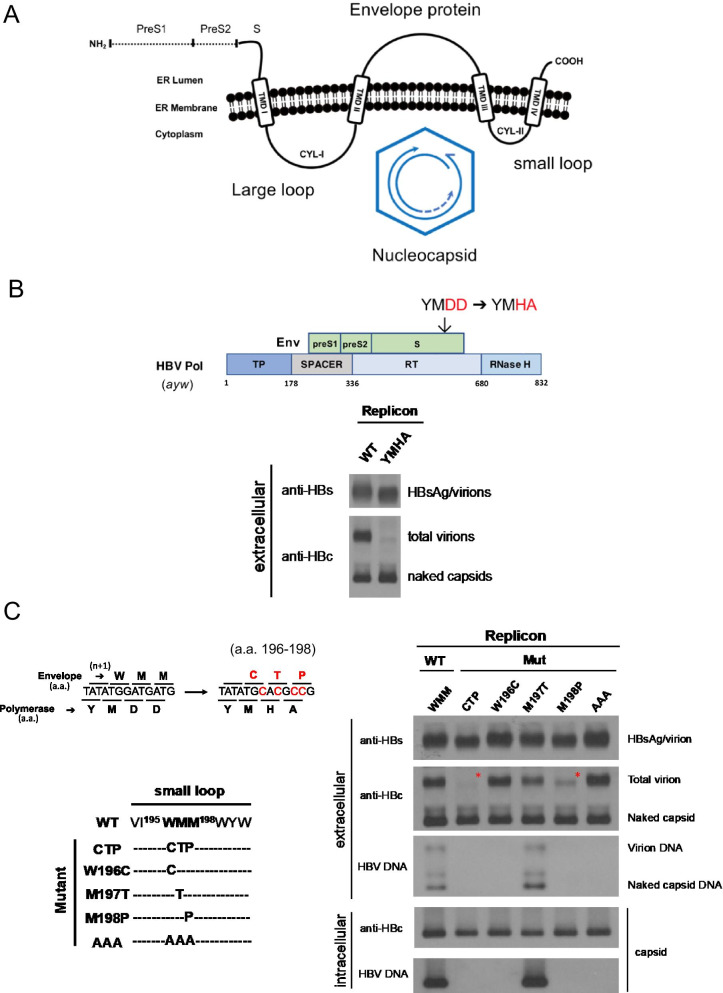
HBV virion secretion is blocked by mutations within a cytosolic small loop of the envelope protein (HBsAg). **A** The predicted topology of the folded HBV envelope protein on the ER membrane contains a cytosolic large loop CYL-I and a small loop CYL-II. A conceptual model for HBV virion assembly and secretion postulates a matrix role for these two cytosolic loops in their interactions with nucleocapsids. **B** A motif YMDD in the wild type reverse transcriptase (RT) domain has been engineered into a YMHA motif. Plasmid DNAs of wild type and mutant HBV DNAs were transfected into a human hepatoma cell line HuH-7. This YMHA mutant secreted no virions in the medium as detected by native agarose gel electrophoresis and Western blot analysis using an anti-HBc antibody. The polymerase gene overlaps with the envelope gene. **C** The YMHA polymerase mutation caused simultaneous changes of amino acid sequences from WMM to CTP at position 196–198 within the small loop of the envelope protein. The CTP triple mutations were dissociated by analyzing individual mutations 196C, 197T, and 198P, respectively. Right panel: Lack of virion secretion was detected only in the triple mutant CTP and a single proline substitution mutant 198P. Red asterisk * highlights the strongly reduced levels of HBc core protein signal of total virions in mutants CTP and M198P

It is known that both large and small envelope proteins are essential to virion secretion [12]. The fold of the small envelope protein on the membrane predicts the existence of two cytosolic loops [13, 14], designated as CYL-I and CYL-II (Fig. 1A; [15]). In this paper, CYL-II is referred to as a small loop (as opposed to the CYL-I large loop). Previously, deletion of the S protein residues 24–28 at the large loop CYL-I blocked HDV virion secretion, yet, no effect on the secretion of HBsAg subviral particles [16]. Serial deletions by 4–6 amino acids at this CYL-I large loop were shown to block HBV virion secretion [17]. In addition, a single amino acid substitution R79K near the end of the large loop also blocked HBV virion secretion, while the secretion of HBsAg subviral particles was normal. In our earlier studies, a naturally occurring S gene mutation L77R at the large loop CYL-I resulted in more than tenfold reduced HBV virion secretion, and 2.8-fold reduced HBsAg in the medium of transfected HuH-7 cells [18]. Confocal microscopy revealed that the L77R mutant HBsAg are largely accumulated within the ER and Golgi. This phenomenon can be rescued by another naturally occurring mutation W74L in the same large loop. Taken together, the large loop of the S protein appears to play a role in virion secretion of both HDV and HBV.

The small loop CYL-II is located near the C-terminus of the S protein (Fig. 1A). Site-directed mutagenesis at CYL-II indicated that the assembly of stable HDV particles is dependent on the small loop, including a tryptophan-196 [19]. Indeed, a tryptophan-rich motif (W196, W199, and W201) in the small loop were shown to have a matrix-like function for S-HBsAg interaction with the large delta antigen (L-HDAg) in HDV assembly [20]. However, unlike HDV, this tryptophan-rich motif in the small loop were dispensable for both assembly and infectivity of HBV virions [15]. In another study, the disparity between HBV and HDV in the requirements of the small loop in virion assembly and secretion, was confirmed [21]. Triple alanine substitutions at the small loop affected no HBV secretion of either genome-free empty virions or genome-containing virions. In literature, unlike the large loop, the small loop of HBsAg is required in virion secretion only for HDV, but not HBV.

In this study, amino acid substitutions at residue 196 or 198 in the small loop altered HBV genome-containing virion secretion. It is surprising to note here that the same proline substitution at a neighboring position 197, adjacent to 196 and 198, exhibited no phenotype in virion secretion. Using a small loop mutant M198P defective in virion secretion, we conducted a complementation experiment, and demonstrated that it is the small S envelope protein, but not L and M, that can successfully restore virion secretion. It merits further investigation in the future on HBV morphogenesis and virion secretion.

## Materials and methods

### Cell line and transfection

HuH-7 cells were maintained in DMEM medium (Invitrogen) supplemented with 10% fetal bovine serum (Gibco), 100 U/ml penicillin and 100 μg/ml streptomycin. HepG2-NTCP cells were generated by NTCP expressing lentivirus and maintained in DMEM medium (Invitrogen) supplemented with 10% fetal bovine serum (Gibco), 100 U/ml penicillin, 100 ug/ml streptomycin and 20ug/ml Blasticidin (Invivogen). Cells were transfected with plasmids by PolyJet™ In Vitro DNA Transfection Reagent (Signagen) according to the Manufacturer's instructions.

### Plasmids

A wild type HBV replicon plasmid, pCHT-9/3091, contains a 1.1-mer HBV genome (*ayw*). Plasmid pCHT-9/3091-pol-YMHA is defective in viral DNA synthesis due to a YMHA mutation at the catalytic site of the reverse transcriptase domain. A pol-null replicon was derived from pCHT-9/3091 [22], with a methionine initiation codon mutation from ATG to ACG [23, 24], and the second methionine codon mutation from ATG to TAG. Plasmid pMT-pol is a WT polymerase expression vector [25]. Single small loop mutations, W196C, M197T, and M198P, were introduced individually into the envelope ORF in pCHT-9/3091. Proline substitution mutants W196P, M197P, and M198P, were engineered by introducing a proline into the envelope ORF of the pol-null replicon. Similarly, envelope mutations W196F, W196S and W196L were introduced into the pol-null replicon plasmid. The L, M, and S envelope expression vectors were engineered using the pCHT-9/3091-Core null backbone. Methionine initiation codons of L, M or S envelope proteins were changed individually from ATG to ACG without affecting the amino acid sequences of the overlapping polymerase ORF. Site direct mutagenesis was performed by using a QuikChange Lightning Site-Directed Mutagenesis Kit (Agilent).

### Native agarose gel electrophoresis for western and southern blot analysis

Culture medium and cell lysates of plasmid-transfected HuH7 cells were collected at 5 days post-transfection. HBV particles in the medium were precipitated by incubation overnight with 10% PEG-8000. PEG precipitates were resuspended in TNE buffer (20 mM Tris pH 7.5, 150 mM NaCl, 1 mM EDTA). Aliquotes of viral and subviral particles from cell lysates and media were resolved by 1% native agarose gel electrophoresis, followed by gel transfer to either nitrocellulose membrane (Amersham) for Western blot, or N + membrane (Invitrogen) for Southern blot analysis. Detailed procedures of Western and Southern blot analyses were as described elsewhere [26, 27]. Rabbit anti-core antibody was diluted 1:5000 for Western blot analysis as described elsewhere [28, 29]. HBsAg/virions was detected with goat anti-HBs antibody (Dako) (1:5000). The digoxygenin-labeled full-length HBV specific DNA probe was used for HBV DNA hybridization in Southern blot analysis.

### Immunofluorescence analysis (IFA)

HBV plasmid-transfected HuH-7 cells were seeded onto glass coverslips (18 mm) in 6-well plates before fixation with 4% paraformaldehyde at 48 h post-transfection. Immunostaining was performed as described [28, 29]. HBV core protein was stained with a rabbit anti-HBc polyclonal antibody (1:2000). HBsAg was stained with a goat anti-HBs antibody (Dako) (1:200).

### Immunoprecipitation and western blot assay

HBV plasmid-transfected HuH-7 cells and HepG2-NTCP cells were seeded in a 6 well plate around 16 h before transfection. Transfected cells were lysed at 5 day post-transfection. Procedures of immunoprecipitation and Western blot assays were performed as described previously [28]. For HBsAg immunoprecipitation, rabbit anti-HBs antibody (Novus) (4 μg) was incubated overnight at 4 °C with protein G-coated magnetic beads. In Western blot analysis, HBV core protein was visualized with an anti-HBc antibody (1:2000), while HBsAg was visualized with a horse anti-HBs antibody (Abcam) (1:1000). Large and middle envelope proteins were detected with a mouse anti-preS2 antibody (Institute of Immunology, Japan) (1:1000).

### Protein topology prediction

The topology of the folded small S protein on the membrane were predicted by the software TMHMM v2.0 (http://www.cbs.dtu.dk/services/TMHMM/).

### Quantification of HBV virion DNA

HuH-7 cells were seeded in a 6-well plate around 16 h before transfection. Supernatants were collected at 5 day post-transfection. HBV virions were immunoprecipitated using a rabbit anti-HBs antibody (Novus) and protein G-coated magnetic beads at 4 °C overnight. Immunoprecipitates were resuspended in TNE buffer for nuclease digestion of input plasmid DNA at 37 °C for 3 h. Viral DNA was extracted by using the High Pure Viral Nucleic Acid Kit (Roche). HBV virion-associated DNA was quantified by qPCR using HBV core-specific primers: forward 5′ GAGTGTGGATTCGCACTCC 3′ and reverse 5′-GAGGCGAGGGAGTTCTTCT 3′ and power SYBR green master mix (ThermoFisher) on Quantstudio 5 (Applied Biosystems). Calculation of HBV genome equivalent (GE) was based on a standard curve of serially diluted HBV replicon plasmid, pCHT-9/3091. Statistical analyses were performed by the GraphPad Prism software. Statistical difference in GE was analyzed by one-way ANOVA test. ***P < 0.001; **P < 0.01; *P < 0.05.

## Results

### No virion secretion of polymerase mutant YMHA

A predicted topology of HBV envelope protein (HBV surface antigen, HBsAg) on the membrane is illustrated in Fig. 1A [13, 14]. There are two cytosolic loops designated as CYL-I (large loop) and CYL-II (small loop) [15]. A conceptual model for virion assembly and secretion had been tested previously [15–17, 19, 20]. This model envisioned that these two loops might serve as a matrix loop in bringing together HBsAg and HBV nucleocapsids or HDV ribonucleoprotein (RNP) complex. As shown in Fig. 1B, we characterized an HBV polymerase (pol) mutant, changing from YMDD to YMHA in the reverse transcriptase (RT) domain. Using the native agarose gel electrophoresis, we discovered by serendipity that mutant YMHA exhibited no detectable virion-associated core protein signal in the medium of HuH-7 cells transfected with the pol-RT mutant YMHA. In contrast, both HBV surface protein antigen and naked capsids can be secreted in a normal manner indistinguishable from the wild type HBV control.

### Polymerase mutant YMHA contains concurrent envelope mutations

Because of the overlapping ORFs between pol and HBsAg (Fig. 1B), this YMHA mutant contains concurrent mutations in the envelope ORF, changing from WMM to CTP at amino acid 196–198 (Fig. 1C). Topologically, these CTP mutations happen to fall within a known small cytosolic loop. To find out which specific amino acid change of these three clustering CTP mutations is responsible for the lack of virion secretion, we compared virion secretion among single envelope mutations W196C, M197T, and M198P (left panel, Fig. 1C). In HBV patients and cell culture, a large excess of empty virions are secreted over the genome-containing virions [5–11]. Only the triple mutant CTP and the single mutant M198P are severely deficient in virion secretion (red asterisk, right panel, Fig. 1C). While mutant M197T has a moderate reduction in empty virion secretion, triple mutant AAA and single mutant W196C exhibited a level of empty virion secretion similar to that of wild type (WT) HBV. There was no apparent difference in the amount of intracellular core protein or extracellular HBsAg and naked capsids (right panel, Fig. 1C). Viral DNA synthesis can only be detected in mutant M197T by Southern blot analysis. The rest of mutants displayed no HBV DNA signal due to their concurrent envelope mutations.

### Cytoplasmic distribution of HBsAg

**Fig. 2:**
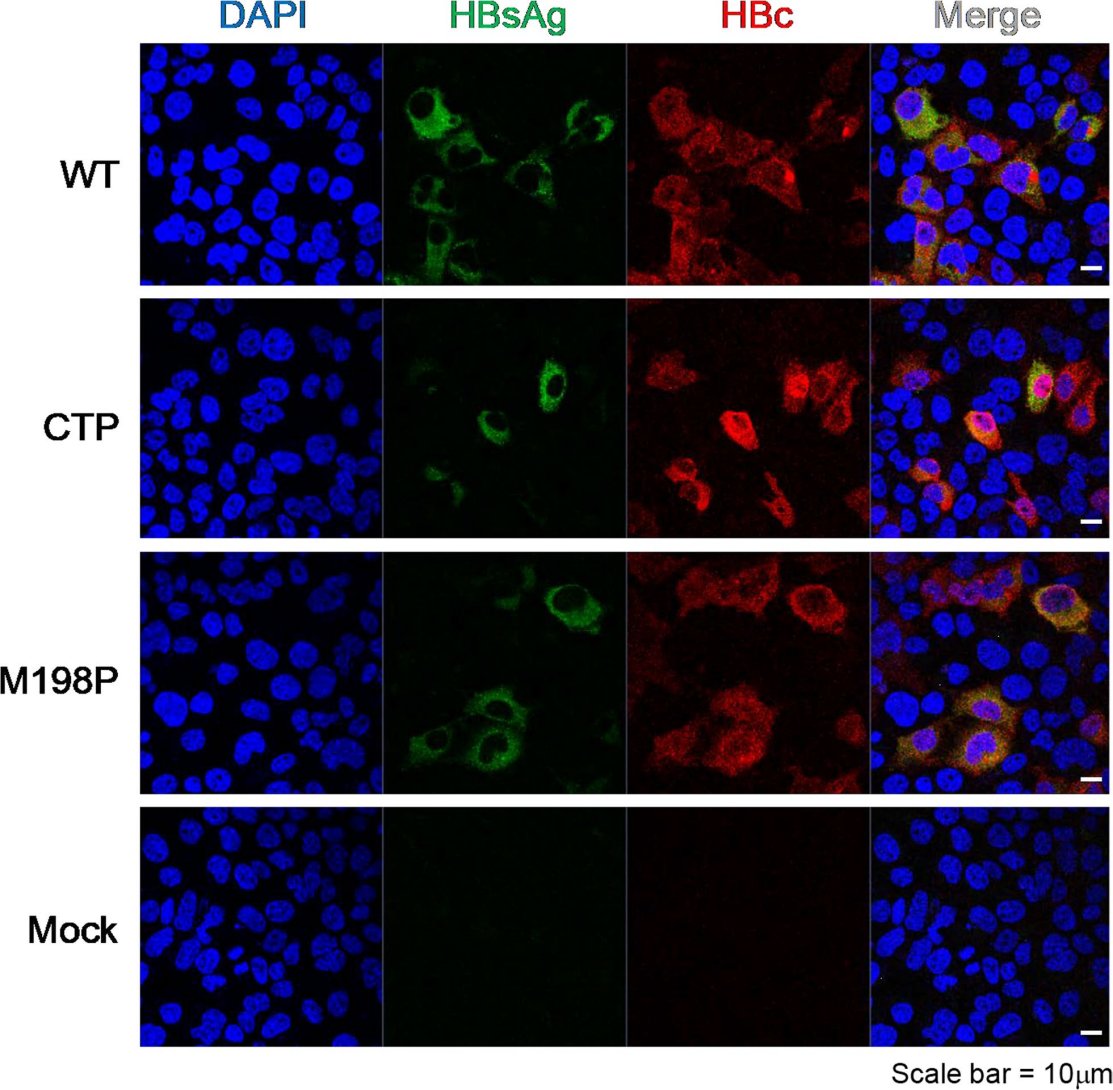
No apparent difference in the subcellular distribution of HBV envelope HBs and core protein HBc between wild type HBV and mutants containing a proline substitution at the small loop of HBs. The intracellular distributions of HBs and HBc were examined by confocal microscopy using anti-surface antibody (green) and anti-core antibody (red)

**Fig. 3:**
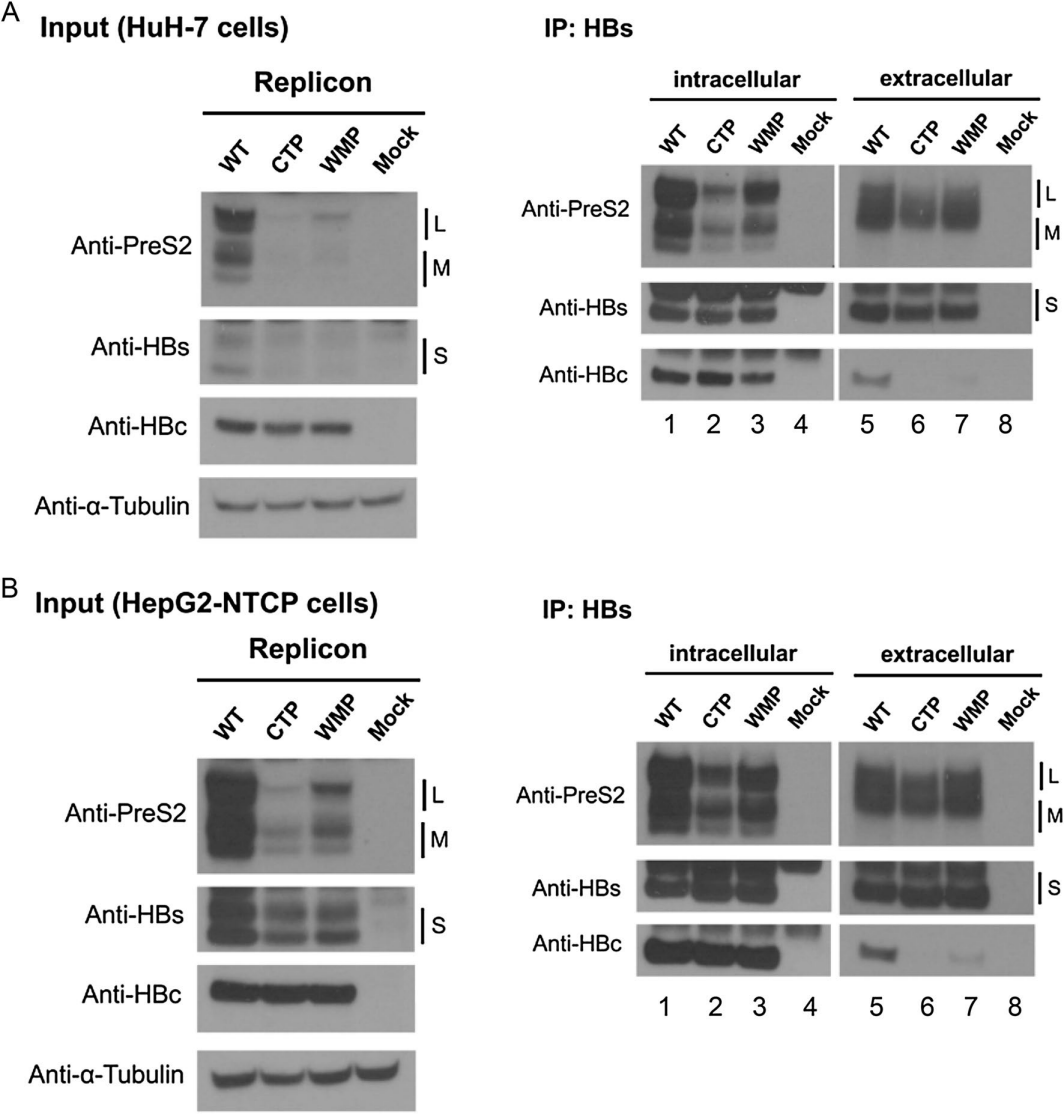
Similar core-envelope interactions were detected between wild type and small loop mutants in HuH7 cells and HepG2-NTCP cells by immunoprecipitation assay and Western blot analysis. **A**, **B** Bead-associated anti-HBs antibody was used to immunoprecipitate intracellular lysate, followed by Western blot analyses using anti-preS2, anti-HBs and anti-core antibodies. Intracellular input. **B** While intracellular core protein signals were similar between WT and mutants (lanes 1–3), no core protein signal and virion secretion were observed in the extracellular compartment (lanes 6–7). L, M, and S: large, middle and small envelope protein

Next, we examined the intracellular distribution of HBsAg between the small loop mutants and wild type HBV by using confocal microscopy (Fig. 2). We detected no apparent difference in the subcellular envelope protein HBs and core protein HBc between wild type HBV, triple mutant CTP and single mutant M198P. It is also tempting to speculate that the small loop mutations could affect the core-envelope interaction leading to abnormality in virion morphogenesis and secretion. To address this issue, we conducted immunoprecipitation assay followed by Western blot analysis. As shown in Western blot of Fig. 3A, relative to the WT control, mutants CTP and M198P had reduced levels of small, middle and large envelope proteins, despite their respective core protein levels are similar to each other.

### Core-envelope interactions

In Fig. 3, anti-HBs antibody was used to immunoprecipitate the intracellular lysates of HuH-7 (Fig. 3A) and HepG2-NTCP (Fig. 3B) cells transfected with wild type and small loop mutants. As expected, in the extracellular compartment, while envelope proteins can be detected in WT and proline substitution mutants by Western blot analysis, we detected no core protein signal in mutants (lane 6 and 7, Fig. 3A and B), indicating the lack of virion secretion. In the intracellular compartment, we detected equal intensities of core protein signal (lane 1–3, Fig. 3A and B), suggesting that core-envelope interaction could be normal for mutants CTP and M198P.

### A nuanced position effect in the small loop on genome-containing virion secretion

**Fig. 4:**
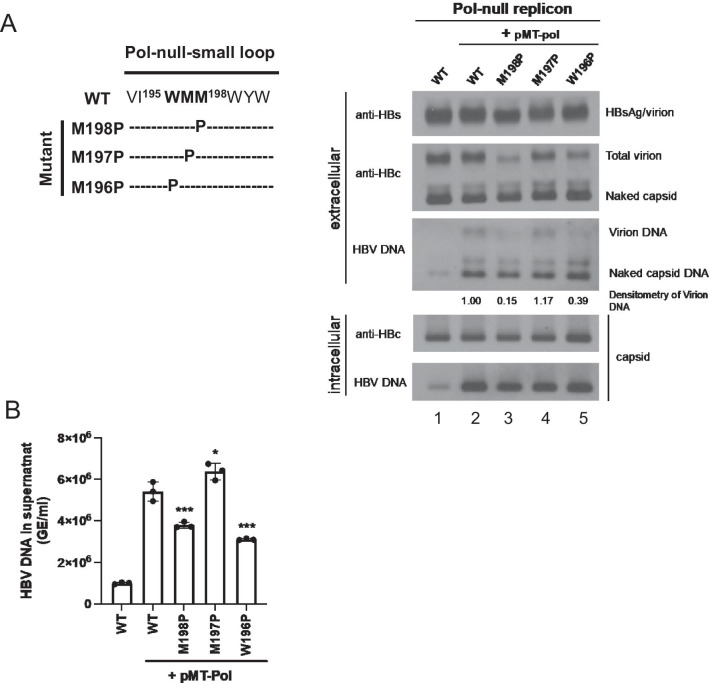
Secretion of genome-containing virions is also affected by proline substitution in the small loop at amino acid 196–198 in a position-dependent manner. **A** HuH-7 cells was cotransfected with a polymerase-deficient replicon plasmid and a wild type polymerase expression vector (pMT-pol). Surprisingly, unlike mutants 196P and 198P, a proline substitution at amino acid 197 resulted in no virion secretion deficiency. Secreted viral particles in the media were enriched by PEG before native agarose gel, followed by Western and Southern blot analyses. Lane 1: A very faint and weak HBV DNA signal can still be visualized from the Pol-null replicon in both intracellular and extracellular panels. Under the strong CMV promoter in the context of pCHT-9/3091 (Materials and Methods), this Pol-null replicon is a bit leaky in polymerase expression and viral DNA synthesis. The virion-associated DNAs were quantified by densitometry and Image J software. **B** Virion-associated HBV DNA genome in the supernatant of HuH-7 cells was analyzed by qPCR. HBV virions were immunoprecipitated with a rabbit anti-HBs antibody and treated with nuclease digestion before viral DNA extraction. HBV DNA signals of small loop mutants cotransfected with pMT-Pol were compared individually to the WT-HBV control. GE: genomic equivalent. Bar graph statistics by one-way ANOVA tests. ***P < 0.001; *P < 0.05

Based on the results In Figs. 1, 2, 3, the polymerase mutant YMHA (HBsAg mutant CTP) is defective in viral DNA synthesis, and its genome-free (empty) virion secretion is arrested by a proline substitution at the small loop. We asked if a similar phenomenon can be extended to secretion of genome-containing virions. We designed a cotransfection experiment by using a pol-null replicon and a wild type polymerase expression vector pMT-pol. In this experiment, we also asked whether the same phenotypic defect in virion secretion can be observed when proline was introduced into positions other than amino acid 198. As shown in Fig. 4A, left panel, we engineered three replicons containing single mutation W196P, M197P, and M198P in the small loop of the HBsAg envelope. By native agarose gel, followed by Western and Southern blot analyses, we observed again significant reduction in the secretion of virion-associated core protein and virion-associated viral DNA of mutant W196P and M198P (lane 3 and 5, Fig. 4A, right panel). Similar results of virion secretion were confirmed by using qPCR analysis (Fig. 4B). Interestingly, in lane 4, the core protein and viral DNA phenotypes of mutant M197P are almost indistinguishable from those of WT HBV. None of these mutants, including mutants CTP, M198P, and W196P, are predicted to have altered the loop size of CYL-II by bioinformatic analysis [software TMHMM v2.0 (http://www.cbs.dtu.dk/services/TMHMM/)]. Therefore, the proline substitution effect on genome-containing virion secretion appears to be position-dependent within the small loop.

### Drug-resistant polymerase mutant YIDD contains envelope mutations W196L/S

**Fig. 5:**
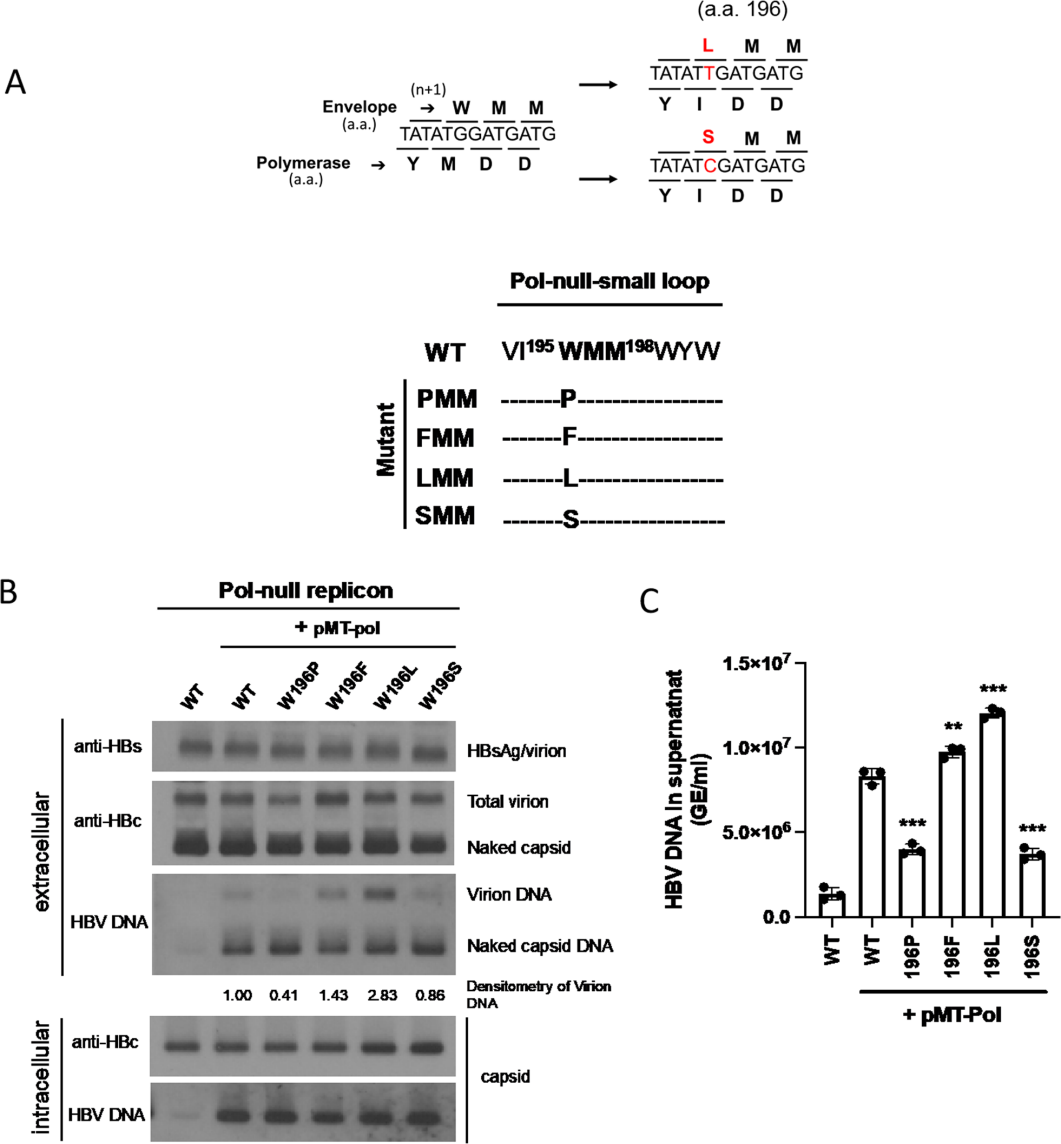
Two lamivudine-resistant polymerase mutants are normal in virion secretion. **A** Polymerase mutants YIDD created corresponding changes in the overlapping envelope ORF—W196L and W196S. **B** No apparent defect in virion secretion was detected in two polymerase YIDD mutants containing envelope mutations W196L and W196S, respectively. Mutant W196L exhibited increased secretion of genome-containing virions. Virion-associated DNAs were quantified by densitometry and Image J software. **C** Virion-associated HBV DNA was analyzed by qPCR as described in Fig. 4B. HBV DNA signals of small loop mutants cotransfected with pMT-Pol were compared individually to the WT-HBV control. GE: genomic equivalent. Bar graph statistics by one-way ANOVA tests. ***P < 0.001; **P < 0.01

Therapeutic treatment of hepatitis B patients with nucleot(s)ide analogs can induce the emergence of drug-resistant polymerase mutants, particularly with amino acid substitutions at the YMDD motif [30–32]. Similar to the polymerase mutant YMHA (Fig. 1C), it is anticipated that corresponding envelope mutations can be generated simultaneously in these drug resistant polymerase mutants. We therefore examined the virion secretion of a highly common polymerase mutant YIDD, which contains corresponding envelope mutations W196L or W196S (from tryptophan to leucine or serine) (Fig. 5A). While the lamivudine-resistant mutations corresponding to sW196L/S inhibited secretion of HDV particles [33], it remains unclear if HBV virion secretion is also affected by the drug resistant polymerase mutants. We found no apparent difference in virion secretion between WT-HBV, mutant W196S, and a control mutant W196F (Fig. 5B). Interestingly, we noted that mutant W196L exhibited significantly increased secretion of genome-containing virions. The results in Fig. 5B were confirmed by using qPCR analysis (Fig. 5C).

### The small loop from the small S envelope is important for virion secretion

**Fig. 6:**
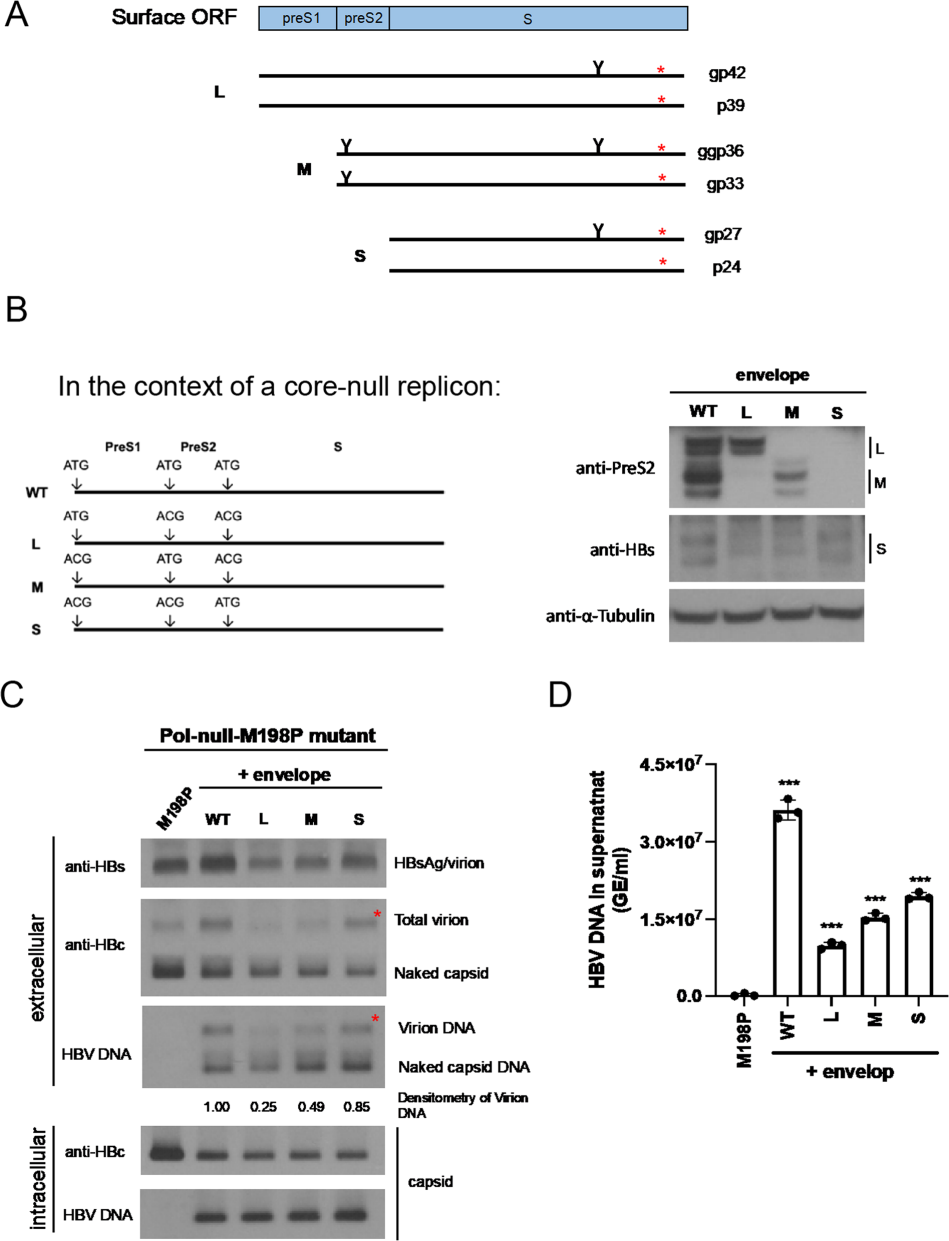
Only the small envelope protein can rescue the virion secretion defect of the proline substitution mutant M198P. **A** A cartoon illustrates that the envelope protein ORF can encode a total of 6 different protein products, including large, middle and small envelope proteins. A proline substitution mutation (red asterisk*) is supposed to be present in all 6 envelope proteins. **B** Western blot analysis detected respective protein products from large, middle and small envelope protein expression vectors. This expression vector is a core-null replicon, which contains no core protein, and is expressing a wild type polymerase and various mutant envelope proteins. Left panel outlines the respective mutations at the initiation codons of large, middle, and small envelope proteins. **C** Cotransfection with the core-null replicon containing large or middle envelope protein did not restore virion secretion. Only the small envelope protein can successfully rescue the virion secretion defect of the small loop mutant M198P. Red asterisk * highlights the HBc core protein signal and HBV DNA signal by complementation with the small S envelope protein. Virion-associated DNAs were quantified by densitometry and Image J software. **D** Virion-associated HBV DNA was analyzed by qPCR as described in Fig. 4B. HBV DNA signals of the complementation experiments were compared individually to the mutant M198P. GE: genomic equivalent. Bar graph statistics by one-way ANOVA tests. ***P < 0.001

HBV envelope protein consists of six family members, including L, M, and S proteins with or without glycosylation (Fig. 6A; [34]). The proline substitution at the small loop is supposed to be present in all members of the L, M, and S envelope proteins. It is therefore unclear which envelope protein member(s) really contributes to the lack of virion secretion. We engineered three different envelope protein expression vectors in the core-null replicon context containing a wild type polymerase. These envelope expression vectors can express only one of the three L, M, or S envelope protein individually by Western blot analysis (Fig. 6B). Next, we performed complementation assay by cotransfecting HuH-7 cells with each of these three envelope protein expression vectors and the pol-null M198P plasmid (Fig. 6C). The defect in genome-containing virion secretion of mutant M198P can be successfully rescued only by cotransfection with the small S envelope expression vector, but not by the L and M expression vectors. Virion-associated viral DNA genome was also rescued simultaneously by the wild type polymerase from the small S envelope expression vector. In the intracellular compartment, cotransfection with different L, M, and S expression vectors produced the same levels of core protein and viral DNA (Fig. 6C, bottom). The Southern blot results in Fig. 6C were confirmed by qPCR analysis (Fig. 6D).

## Discussion

HBV virion morphogenesis requires the productive interaction between nucleocapsid particles and the envelope protein(s). Neither HDV nor HBV is known to have a matrix protein which can specialize in facilitating the envelopment of nucleocapsids or an RNP complex. It is tempting to hypothesize that these two cytosolic loops (CYL-I and CYL-II) may serve as a binding interface between envelope proteins on the ER membrane and the nucleocapsids in the cytoplasm. We revisited this hypothesis by focusing on the small loop CYL-II of HBsAg. In previous studies, this small loop had been shown to have a function in virion secretion for HDV, but not HBV [15, 20, 21]. Here, it is surprising that a proline substitution at amino acid 196 or 198 resulted in significantly diminished secretion of virions with (Figs. 4, 5, 6) or without (Fig. 1) containing an HBV genome. If CYL-II is nothing but a small loop without any important structure and function, it is unclear why only W196P and M198P had a phenotypic effect on virion secretion, but not M197P. This position-dependent effect of proline substitution on virion secretion argues for a nuanced role of the small loop in virion morphogenesis and secretion.

The significant reduction in the intracellular HBsAg protein (Fig. 3) in the small loop mutants is not caused by a reduced level of HBsAg mRNA (Additional file 1: Fig. S1). Instead, it could be caused by a post-transcriptional effect, such as protein stability. Given the deceased intracellular level of HBsAg (Fig. 3), it is unclear why the small loop mutants exhibited a similar level of core-envelope interaction complex in the IP-Western blot experiment (Fig. 3). One possibility is that HBsAg particles (22 nm) is well known to exist in large excess over the virion particles (42 nm). Only a very small amount of HBsAg is needed for virion assembly and morphogenesis. Therefore, the reduction in the intracellular HBsAg level had no significant effect on the core-envelope interaction. Alternatively, since small loop mutants were unable to secrete virions to the extracellular compartment (Fig. 1B and C), the intracellular core-envelope complex was accumulated to a higher level accordingly. In this scenario, the HBsAg reduction effect on the core-envelope interaction is compensated by the accumulation of core-envelope complex arrested in the virion secretion pathway. Finally, the possibility that the mutant core might interact better with the envelopment machinery cannot be excluded. One caveat here is that better binding between core and envelope cannot necessarily be equated to better virion secretion.

Our complementation assay demonstrated that the small loop of the small S envelope protein, but not the large L and middle M envelope protein, is required for virion secretion (Fig. 6). In Fig. 6C, the middle form of HBsAg (M) indeed exhibited very weak signal of rescue. However, it is well known that the M envelope protein is not essential for HBV replication and virion secretion in cell culture [12, 35]. Although the small loop of the L envelope protein is not required for virion secretion, the L envelope protein itself is essential to HBV virion morphogenesis and secretion [12]. By serial deletions of the L protein, a short C-terminal region near the end of the preS1 domain, including arginine-103 to serine-124, was shown to be required for virion maturation [36, 37]. The interaction between the core protein of nucleocapsids and the preS1 domain of the L envelope protein was also demonstrated in another experimental setting. Naturally occurring core mutants I97L (isoleucine to leucine) or F97L (phenylalanine to leucine), exhibited a so-called “immature secretion” phenotype, which allows excessive secretion of virions containing an immature genome [38–41]. A pre-S1 envelope mutation A119F, changing an alanine (A) to a phenylalanine (F), can erase the immature secretion phenotype of the mutant I97L and successfully restore the wild-type-like selective export of the mature genome [42]. Altogether, both L and S envelope proteins are required for virion assembly and secretion.

Longer term treatment of chronic hepatitis B patients with nucleot(s)ide analogs can result in the emergence of drug-resistant polymerase mutants, for example, changing from YMDD to YIDD at the RT domain [30–32]. In theory, these kinds of drug-resistant variants should have no problem in virion secretion. Otherwise, they could not have amplified themselves and eventually evolved into a predominant species in treated patients. However, over the past few decades, there is no direct quantitative measurement of the virion secretion efficiency of drug-resistant polymerase variants. In our study, we noted that the polymerase YIDD mutant with a W196S envelope mutation has no apparent defect in HBV virion secretion (Fig. 5B). However, we were intrigued that the same polymerase mutant YIDD with a different envelope mutation W196L, displayed an even stronger signal of virion-associated DNA, at least in this in vitro HuH-7 cell culture setting. The enhanced virion secretion of mutant W196L provides another evidence that the small loop of the small envelope protein could be engaged in productive core-envelope interaction and virion morphogenesis. As a side note, it is worth mentioning here that neither envelope W196L nor W196S could support HDV virion secretion [33]. Altogether, these virion secretion results could explain the emergence of drug resistant HBV polymerase mutants in patients receiving nucleos(t)ide analog therapy.

A number of host factors involved in HBV particle release have been reported. The Endosomal Sorting Complex Required for Transport (ESCRT) is a house-keeping machinery for both intracellular sorting and trafficking of ubiquitinated protein cargos. Vps4 is a cellular ATPase which is often associated with ESCRT-0 in membrane dynamics. HBV replication and virion secretion can be significantly inhibited by Vps4 dominant negative and ATPase-defective mutants [43, 44]. ESCRT-II, ESCRT-III, Vps4, and gamma 2-adaptin had been reported to be involved in HBV morphogenesis and virion secretion [44–46]. Aberrant expression of HGS (over- or under-expression) of ESCRT-0 can inhibit HBV replication, increase the release of naked capsids, yet reduced the extracellular virions and HBsAg subviral particles [27]. We assumed here that the small loop mutation does not alter the cellular HGS expression level which could then affect virion secretion. Furthermore, it should be noted that the inverse correlation between secreted virions and secreted naked capsids in the HGS studies [27] cannot be extended to our current studies on the envelope small loop. As shown in Figs. 1B, C, 4A, 5B, and 6C, secreted naked capsids have no inverse correlation with secreted virions. In addition, we observed no increased accumulation of intracellular capsid protein and intracellular capsid-associated HBV DNA in the small loop mutants with reduced virion secretion.

Most recently, ERGIC-53, a cellular high-mannose specific membrane lectin, and the endoplasmic reticulum (ER) export machinery COPII (coat protein complex II), were shown to be important for HBV trafficking and egress [47]. COPII subunits Sec24A, Sec23B, and Sar1 are required for release of both viral and subviral particles. ERGIC-53, Sec24A, and ESCRT, appeared to be involved later in virion secretion after envelopment of nucleocapsids. To date, it remains unclear if CYL-I and CYL-II can bind to any cellular factors involved in the earlier stage of virion morphogenesis and secretion. We speculated here that our current study on the small loop is more related to the early event in virion assembly and morphogenesis in ER/Golgi, while the studies on the ESCRT machinery is more related to the late event in virion secretion in MVB by energy-dependent membrane dynamics.

## Conclusions

We revisited the relationship between HBV virion secretion and the small loop of the small envelope protein. Amino acid substitutions at certain positions within the small loop could affect the egress of both empty virions and genome-containing virions. Understanding better the mechanism of HBV virion secretion could contribute to the development of HBV curative therapy.

## Supplementary Information

**Additional file 1.** Similar levels of HBV RNAs were detected between wild type and small loop mutants by Northern blot analysis.

## Data Availability

All data generated in this study are included in this manuscript.

## Abbreviations

HBV: Human hepatitis B virus
HDV: Hepatitis delta virus
HBsAg: Hepatitis B surface antigen
HBc: HBV core protein
Pol: HBV polymerase
RT: Reverse transcriptase domain of pol
ORF: Open reading frame
L: Large envelope protein
M: Middle envelope protein
S: Small envelope protein
CYL-I: Large cytosolic loop I
CYL-II: Small cytosolic loop II
ESCRT: Endosomal Sorting Complex Required for Transport
HGS: Hepatocyte growth factor-regulated tyrosine kinase substrate
COPII: Coat protein complex II
Sec24A Sec 23B Sar1: COPII subunit
ERGIC-53: An ER-Golgi intermediate compartment (ERGIC) marker protein
