# Supplementary material for: A nuanced role of the small loop of hepatitis B virus small envelope protein in virion morphogenesis and secretion
Source: J Biomed Sci. 2021 Dec 1;28:82. doi: 10.1186/s12929-021-00780-0 (PMC8638367; doi:10.1186/s12929-021-00780-0)
Supplement: Supplementary file 1 — Additional file 1. Similar levels of HBV RNAs were detected between wild type and small loop mutants by Northern blot analysis. [file 12929_2021_780_MOESM1_ESM.docx]

**Supplementary Information**

**A nuanced role of the small loop of hepatitis B virus small envelope protein in virion morphogenesis** **and secretion**

Short title: HBsAg small loop in virion egress

Chih-Hsu Chang^1, 2^, Shu-Fan Chou^3^, and Chiaho Shih^2,4*^

^1^Graduate Institute of Microbiology, College of Medicine, National Taiwan University, Taiwan.

^2^Institute of Biomedical Sciences, Academia Sinica, Taipei, Taiwan.

^3^Department of Microbiology, Harvard Medical School, Boston, Massachusetts, USA

^4^Graduate Institute of Medicine, Kaohsiung Medical University, Kaohsiung, Taiwan

* Corresponding author

Mailing address: Institute of Biomedical Sciences, Academia Sinica, Taipei, Taiwan

Tel: 886-2-2652-3996; E-mail: cshih@kmu.edu.tw

**Supplementary Methods**

**Northern blot analysis**

The RNA samples of HBV plasmid-transfected HuH-7 cells and HepG2-NTCP cells were extracted by TRIzol reagent (Ambion) at 5 days post-transfection. The RNA samples (15μg each) were subjected to 1.2% formaldehyde agarose gel electrophoresis, followed by gel transfer to N+ membrane (Invitrogen) for Northern blot analysis. Standard procedures of Northern blot analysis were as described previously (1). Hybridization of HBV RNAs was probed with a digoxygenin-labeled full-length HBV DNA. 28S and 18S ribosomal RNAs were stained with HealthView Nucleic Acid Stain (Genomics).

**Supplementary Figures**

**Fig S1.** Similar levels of HBV RNAs were detected between wild type and small loop mutants by Northern blot analysis. **A & B**) HBV RNA signals were similar between WT and mutants in HuH-7 cells (lanes 1-3) and HepG2-NTCP cells (lanes 5-7). Upper panel: Major HBV RNA species include the 3.5 kb pregenomic RNA and the 2.4/2.1 kb mRNA of HBsAg. Lower panel: Ribosomal RNAs were included as an internal control for sample loading.

**Supplementary Reference:**

1. Yang CC, Huang EY, Li HC, Su PY, and Shih C. Nuclear export of human hepatitis B virus core protein and pregenomic RNA depends on the cellular NXF1-p15 machinery. PLoS ONE 2014; 9(10): e106683.
